# Chromosome-scale telomere to telomere genome assembly of common crystalwort (*Riccia sorocarpa* Bisch.)

**DOI:** 10.1038/s41597-025-04373-6

**Published:** 2025-01-15

**Authors:** Katarzyna Krawczyk, Joanna Szablińska-Piernik, Łukasz Paukszto, Mateusz Maździarz, Paweł Sulima, Jerzy Andrzej Przyborowski, Monika Szczecińska, Jakub Sawicki

**Affiliations:** 1 Department of Botany and Evolutionary Ecology, University of Warmia and Mazury in Olsztyn, Plac Łódzki 1, Olsztyn, 10-719 Poland; 2 Department of Genetics, Plant Breeding and Bioresource Engineering, University of Warmia and Mazury in Olsztyn, Plac Łódzki 3, Olsztyn, 10-724 Poland

**Keywords:** Plant genetics, Plant evolution

## Abstract

*Riccia sorocarpa* Bisch., commonly known as common crystalwort, is a plant belonging to the Marchantiales order with a cosmopolitan distribution among a wide range of habitats: fields, gardens, waste ground, on paths, cliff tops, and thin soil over rocks or by water bodies. However, research into the genetic aspects of this species is limited. In this study, the chromosome-scale telomere-to-telomere genome of *R. sorocarpa* was assembled exclusively by Oxford Nanopore long-read sequencing and Pore-C technology. A high-quality chromosomal-scale assembly was obtained with a final genome size of 376.690 Mbp, contig N50 of 49.132 Mbp and 97.02% of the assembled contigs associated with the eight chromosomes. Genome assembly completeness was confirmed by BUSCO analysis accounting 91.8%. Among 27,626 total genes, 23,562 (85.29%) were functionally annotated. Moreover, collinearity of Marchantiales was analyzed as well as gene family evolution and DNA methylation profile. The availability of this genome, which is the second telomere-to-telomere liverwort assembly, opens up new avenues for in-depth analysis of *R. sorocarpa* genetic diversity and genomic characteristics.

## Background & Summary

Liverworts, members of the division Marchantiophyta, are non-vascular plants representing an early-diverging lineage in the evolution of land plants. Fossil records indicate the emergence of liverworts over 480 million years ago, suggesting their critical involvement in the initial colonization of land by plants^1^. Their gametophyte-dominant life cycle, where the haploid stage is the conspicuous plant body, offers valuable insights into the evolution of alternation of generations within the plant lineage^2^.

Liverworts present two primary morphologies: thallose, characterized by flattened, lobed structures, and leafy, exhibiting small leaf-like structures along a stem. The presence of specialized oil bodies within their cells is a unique feature of liverworts. These oil bodies contain a variety of terpenoids and aromatic compounds, which are hypothesized to serve protective functions against herbivory^3,4^. Liverworts display both sexual and asexual reproductive strategies, facilitating dispersal and colonization of new habitats^5^. Liverworts occupy a wide range of habitats globally, with distribution contingent upon moisture availability^6^. They are commonly found in humid environments such as tropical and temperate forests, as well as specialized microhabitats like moist rock surfaces.

*Riccia* L. represents the most widespread and diverse genus within the complex thalloid liverworts (Class Marchantiopsida), comprising approximately 240 species^7^. These liverworts exhibit a range of growth habits, forming rosettes or extensive mats on exposed soil, or existing as semi-aquatic floating forms. Several *Riccia* species play a key role within delicate biological soil crusts, dominating as the primary bryophyte of arable fields. They also thrive in proximity to semi-permanent water bodies, on compacted dirt surfaces, and even within urban environments, colonizing bare patches in lawns or cracks in pavement^8^. Riccia produces large, thick-walled spores with intricate ornamentation, and lack both elaters and oil bodies^9^.

*Riccia sorocarpa* Bisch. is a species that’s both easy to identify and incredibly widespread. Its glaucous green color, turning brown with age, is a distinctive trait, but the most defining characteristic is thickness of thallus, which is 1-2 times wider than high. The margins of the thallus are well-defined, rising at an acute angle and lined with tiny, colorless and hyaline scales. common crystalwort has a cosmopolitan distribution, found across much of the globe, including Europe, Asia, Africa, Australia, North America, and South America^10^. This remarkable adaptability allows it to thrive in fields, gardens, waste ground, on paths, cliff tops, and even in thin soil over rocks or by water bodies. It tolerates both acidic and basic soil conditions growing often together with other *Riccia* species like *R. glauca* and *R. subbifurca*. Like other liverworts, it can reproduce both sexually through spore production or asexually by fragmentation. Interestingly, in contrast to model liverwort *Marchantia polymorpha*, *R. sorocarpa* is monoecious, meaning both male and female reproductive structures are found on the same plant. The preliminary phylogenetic studies resolved *R. sorocarpa* as monophyletic, forming common clade with species like *R. nigrella*, *R. macrocarpa*, *R. crinita* and *R. atromarginata*^8^ and later study resolved *R. sorocarpa* as sister to *R. crinita* and *subcrinita*^11^. In comparison to other plant lineages, the nuclear genomes of liverworts are poorly explored, with only one, model species *Marchantia polymorpha* assembled to chromosome scale^12^. The genomes of other species of complex thalloid liverworts were published as pseudochromosome or draft assemblies^13,14^.

Nanopore sequencing stands apart in DNA sequencing with its remarkable capability to produce exceptionally long reads, detect modifications directly on the DNA molecule, and provide data in real-time. Long reads, often reaching hundreds of thousands of bases, offer an unparalleled advantage in tackling repetitive and complex regions of the genome. These long reads revolutionize genome assembly, enabling scientists to overcome the previous limitations of fragmented short-read approaches and unveil the true complexity of genomic architecture along with DNA modifications. Continuous improvements in nanopore sequencing technology have led to the capability of assembling and annotating chromosome-scale genomes using exclusively nanopore sequencing data^15^.

Liverwort genomic resources are still underrepresented, especially in comparison to mosses and vascular plants. Up to the date only two species were sequenced and assembled at chromosome scale: model species *M. polymorpha*^12^ and *Ricciocarpos natans*^16^, while few other species were assembled at scaffold scale including *M. inflexa*, *M. palacea* and *Lunularia cruciata*^13,17^.

To expand knowledge in the field of liverworts genomics, a high-quality chromosomal-scale telomere-to-telomere assembly of *R. sorocarpa* was generated applying exclusively Oxford Nanopore long-read sequencing and Pore-C technology. The assembled genome had a size of 376.690 Mbp with a contig N50 of 49.132 Mbp and GC content 41.53,% (Table 1), and 97.02% of the assembled bases associated with the eight chromosomes. This genome will be a valuable resource as a genetic basis for further studies, including adaptive evolution, phylogenomics, population genomics and many others.

**Table 1:** Assembly statistics.

Genome assembly statistics	
Genome coverage [median]	163
Estimated genome size [Mbp]	320-390
Number of chromosomes	8
Chromosomes N50 [bp]	49,131,805
Longest chromosome [bp]	55,960,836
Number of contigs	3,218
Number of scaffolds	951
Scaffolds N50 [bp]	790,541
Scaffold NG50 [bp]	839,990
Longest scaffold [bp]	3,307,131
Genome size [bp]	376,689,626
Contig N50 [bp]	258,577
Longest contig [bp]	1,529,652
GC content [%]	41.5
Completeness BUSCO [%]	91.8
Single-copy BUSCO [%]	85.1
Duplicated BUSCO [%]	6.70

## Methods

### Material collection and cultivation

**Fig. 1:**
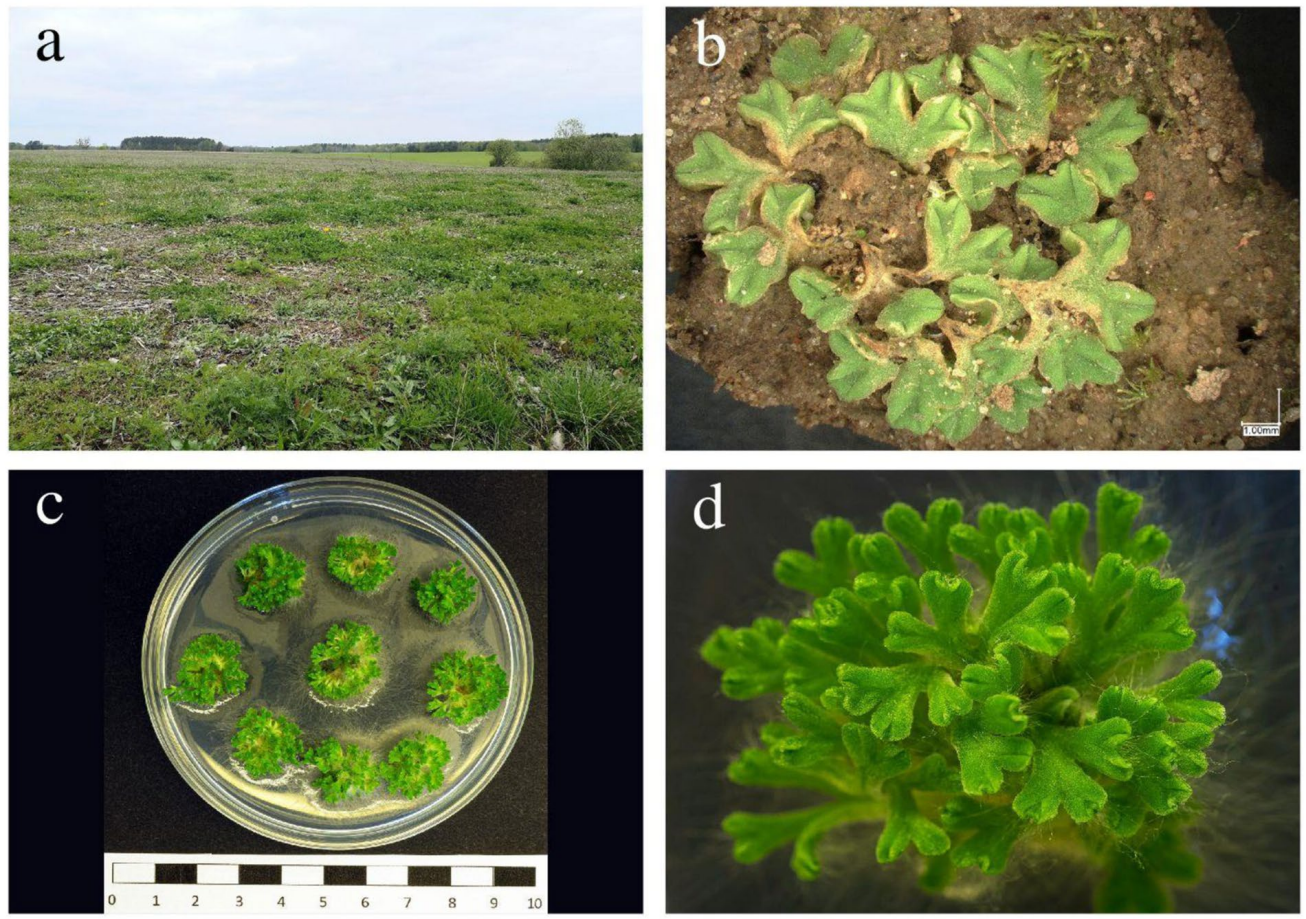
Arable field near Butryny village (NE Poland) - the collection site (**a**). Thalli morphology on *Riccia sorocarpa*. (**b**) *In vitro* culture (**c**) and thalli morphology under in vitro conditions (**d**).

Small, young fragments of *Riccia sorocarpa* plants were collected from an arable field (Fig. 1) near Butryny (NE Poland, 53.603 N, 20.595E). Each fragment was treated as a possible separate plant*. In vitro* cultures of *R. sorocarpa* were carried out in the Department of Genetics, Plant Breeding and Bioresource Engineering of the University of Warmia and Mazury in Olsztyn (Poland). The *R. sorocarpa* fragments were washed in running tap water for 15 minutes. Thereafter, they were surface-disinfected for 10 min. using 0.5% calcium hypochlorite (Chempur, Poland) with addition of 0.05% TWEEN-20 (POCH, Poland). After sterilization the explants were rinsed three times with sterile distilled water for 5, 10 and 15 min. Each *R. sorocarpa* explant was cultured separately on Petri dish with the solid half strength Gamborg’s B5 medium (½ GB5)^18^ including ½ basal salts (Sigma Aldrich Co., USA); organics and vitamins^19^ with 20 g/l sucrose, 8 g/l agar (A&A Biotechnology, Poland) and pH 6.0. Plants were grown in climate chambers at 24 °C under long-day conditions with 16 h and 8 h dark photoperiod. After first passage, the upper fragments of sterile plants of *R. sorocarpa* were used as secondary explants for micropropagation and were placed on the ½ GB5 medium in the form of small clumps separated by approx. 1–2 cm. Micropropagated plants were used in the next parts of this study.

### Long-read DNA sequencing and genome assembly

Before cryo-grinding the plants were kept in the dark for three days to reduce the abundance of secondary metabolites. DNA isolation was performed using DNeasy® Plant Mini Kit (Qiagen) according to the manufacturer’s protocol. Quality and quantity control of the sample was carried out under Oxford Nanopore Technologies (ONT) recommendations. Sample concentration was checked using Qubit^TM^ fluorometer and Qubit^TM^ dsDNA HS Assay Kit and amounted 20 ng/µl, absorbance ratio A_260/230_ and A_260/280_ was checked with Carry spectrophotometer (Agilent) accounting 2.0 and 1.77, respectively. A total of 900 ng of native DNA was used for library preparation with Ligation sequencing kit V14 (LSK-SQK114) and NEBNext® Companion Module for ONT Ligation Sequencing (New England Biolabs). The sequencing experiment was run on the R10.4.1 flow cell (FLO-PRO114M) and the PromethION 2 Solo sequencing device.

**Fig. 2:**
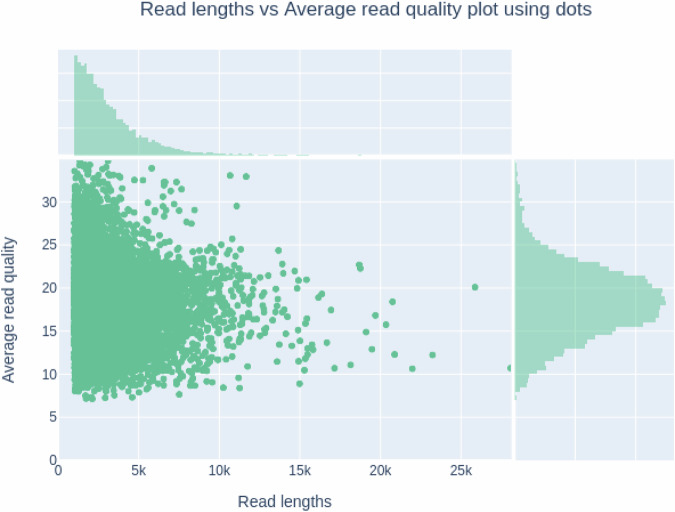
Distribution of ONT DNA read lengths, considering the average read quality.

The nanopore sequencing produced 96.206 Gbp of raw data composed of 40,443,226 long reads. Raw reads were basecalled using standalone Dorado 0.5.1 software (https://github.com/nanoporetech/dorado, ONT) with a super accuracy model (SUP) and stereo-duplex mode. After removing the low-quality reads by Porechop (v0.2.4) (https://github.com/rrwick/Porechop), clean data was evaluated by Whokaryote (v1.1.2)^20^ and Centrifuge (v1.0.4)^21^ software to filter out potential bacterial DNA contamination. Among the 20,079,689 long reads, counting 64.795 Gbp with 3,929 N50 read length, 98.4% indicated the quality of sequence Q > 10 and 80.6% Q > 15 (Fig. 2). A fastq file was used to count K-mer frequencies using the *kmerfreq* program. Subsequently, the genome size was estimated using the *GCE* program and kmerfreq files^22^. The estimated genome size for k-mers from 14 to 21 fell within the 320-390 Mbp range. The preliminary assembly was performed by Flye (v2.9-b1774)^23^ software with 3 rounds of polishing and -genome_size estimation equal to 400 m and–nano-hq parameters. As a result, the draft genome of *R. sorocarpa* was composed of 3,218 contigs with a total sequence length equal to 376,689,626 bp and a contig N50 = 258,577 (Table 1).

### Pore-C procedure and chromosome-scale assembly

Approximately 2 g of fresh weight of the aerial parts of the thallus of *R. sorocarpa* were used for the Pore-C method. The restriction enzyme Pore-C for plant samples protocol (RE-Pore-C, ONT) was applied to capture three-dimensional interactions of DNA within chromatin (crosslinking of chromatin). Briefly, in the first step ‘in-nucleus’ chromatin was captured using formaldehyde to preserve nuclear structures within each chromosome. Then, the crosslinked plant material was cryo-grounded into a fine powder and proceeded to nuclei isolation. The suspension obtained after incubation in the nuclei isolation buffer was passed through a cell strainer (40 μm). The filtrate was purified with a washing buffer and proceeded by chromatin digestion with NlaIII (NEB) restriction enzyme (18 h, 37 °C), which was then deactivated by heat denaturation.

In the next step, a proximity ligation reaction with 40,000 U of T4 DNA ligase (6 h, 16 °C) was performed, followed by protein degradation leading to chromatin de-crosslinking during which the sample was incubated with proteinase K (100 μl) and RNaseA (100 ng/μl) for 18 h (56 °C). The reaction efficiency was increased by two additional rounds of proteinase K digestion (protocol HiPore-C v1)^24^ performed using 10 μl of Proteinase K (20 mg/ml) for each hour of reaction.

Finally, the extraction of DNA with chilled phenol:chloroform:isoamyl alcohol and EDTA was performed. The DNA was then precipitated with NaCl, washed with 80 and 70% ethanol, and eluted in 100 µl TE buffer. Fragment length distribution and DNA integrity were analyzed with TapeStation using genomic DNA ScreenTape Assay (Agilent) reaching maximum intensity peak with length 29,362 bp and DIN 7.1, respectively. The concentration (10.2 ng/μl) was determined using the Qubit fluorometer HS DNA assay kit.

**Fig. 3:**
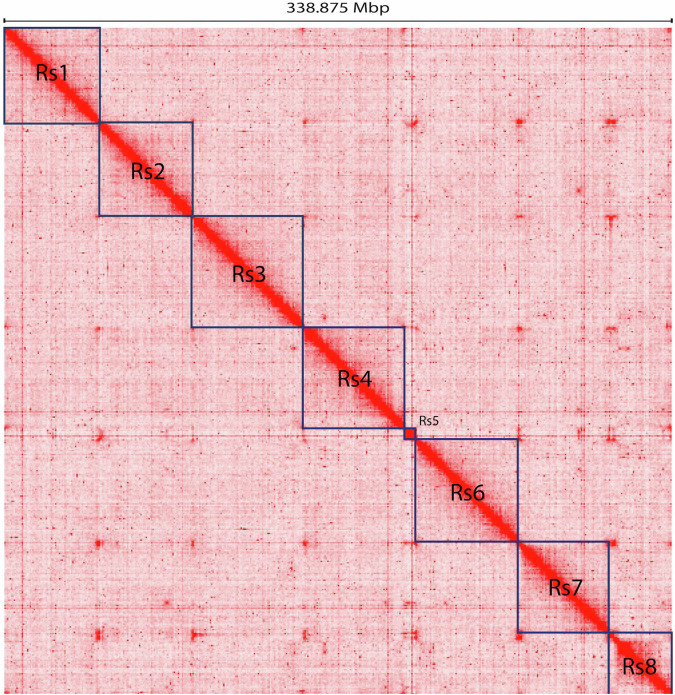
Pore-C interaction heatmap for the *R. sorocarpa* genome.

Two separate libraries were prepared for nanopore sequencing of proximity ligated DNA fragments with Ligation sequencing DNA V14 kit (SQK-LSK-114) according to the manufacturer’s protocol. The only difference was time of end repair (NEBNext Companion Module for ONT Ligation Sequencing) which was extended to 20 min at 20 °C and 20 min at 65 °C. The libraries were sequenced on PromethION P2 Solo using R10.4.1 flow cells (FLO-PRO114M), generating 3,858,602 reads consisting of 12.058 Gb of raw data with a mean quality of 13.7. The trimmed Pore-C sequences were mapped to the draft assembly genome using the Pore-C-Snakemake pipeline (https://github.com/nanoporetech/Pore-C-Snakemake). The Pore-C contacts were generated using the 3D-DNA v.180419 procedure^25^. After all, combining the draft genome and Pore-C contact data was used to create scaffolds by YaHS (v1.2) software^26^. The procedure of Pore-C generation and scaffolding were iterated in the two loops and next the manual curation was done based on a heatmap of pairwise contacts using JuiceBox (v1.11.08)^27^ (Fig. 3). The JuiceBox manual scaffolding result was reviewed using run-asm-pipeline-post-review.sh command implemented in 3D-DNA. Finally, the chromosome sequences were polished to reduce N bases into the *R. sorocarpa* draft genome using TGS-GapCloser software (v1.2.1)^28^. The final genome assembly resulted in eight chromosomes what corresponded with literature data^29^. It yielded a total length of 376,689,626 bp with the largest chromosome length of 55,960,836 bp. The 97.02% contigs were anchored to all chromosomes with an N50 length of 49.132 Mbp. After re-evaluating the primary assembly with ntLink software^30^ 951 scaffolds from the contigs and trimmed Nanopore reads were generated, achieving an NG50 length of 839,990 bp. Evaluating the assembly genome by Inspector software^31^ the genome had a high consensus quality (QV) equal to 35.23 and reads mapping rate equal to 96.98%. To identify the basic repeated telomeric unit, the telomeric-identifier v.0.2.41 (https://github.com/tolkit/telomeric-identifier) was used with default parameters (1% of the length of the chromosome either side). 2031 AAAACT motifs were detected as telomeric repeat units.

### Repetitive sequence and genome annotation

**Fig. 4:**
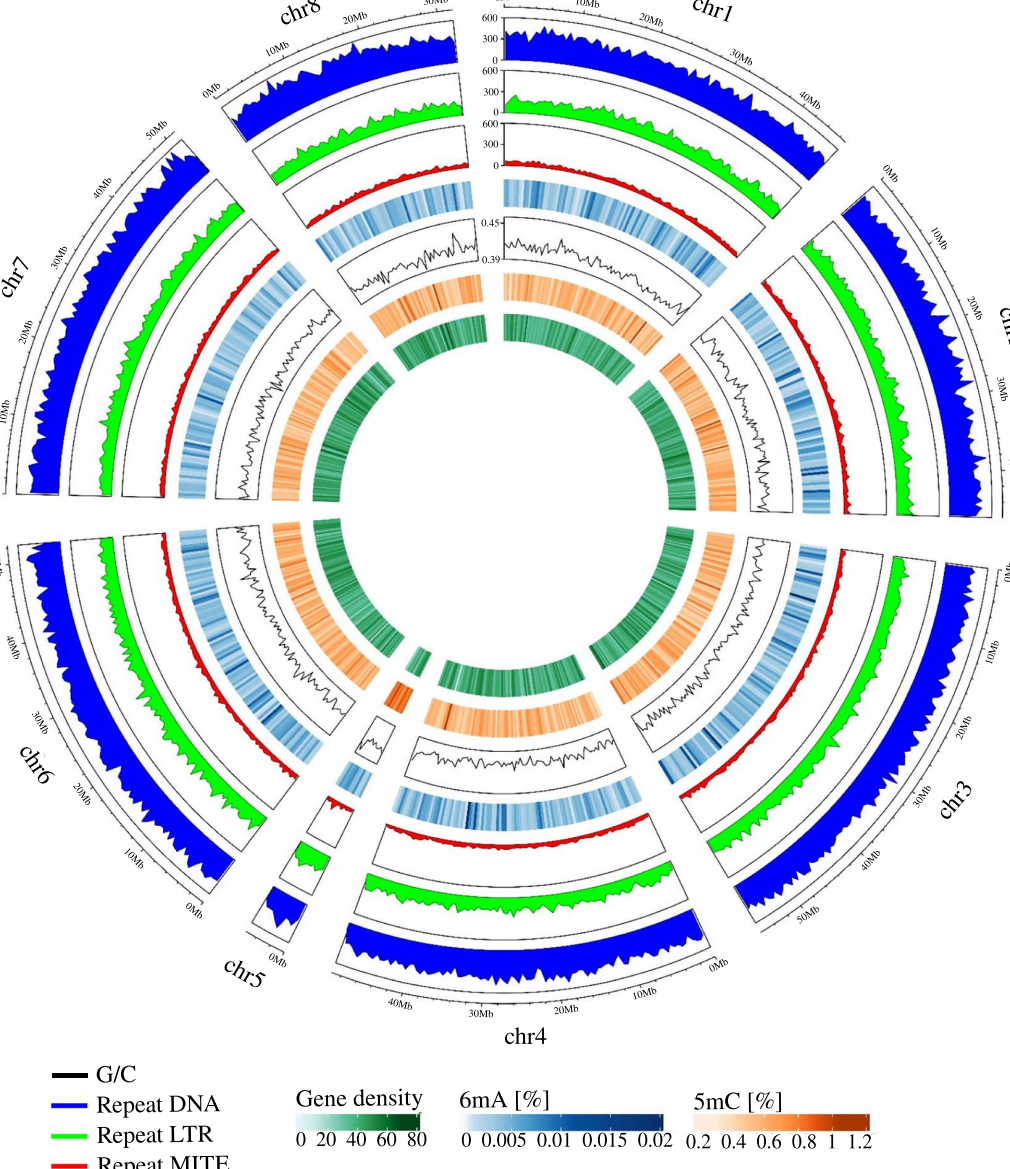
Circos plot of eight *R. sorocarpa* chromosomes. The basic unit of information on the circus is 500,000 nucleotides. The first track (blue) shows the distribution of transposon DNA, the second track (green) shows the distribution of LTR transposons, the third track (red) shows the distribution of MITEs transposons, the fourth track reflects the blue heatmap of % 6mA to all adenines in one window, the fifth track shows the G/C ratio, the sixth track shows the red heatmap of % 5mC to all cytosines in the window, and the last track shows the gene density heatmap in the window.

The chromosome-scale genome was scanned by EDTA^32^ and RepeatMasker (https://github.com/rmhubley/RepeatMasker?tab=readme-ov-file#repeatmasker) tools and repetitive sequences accounted for 42.17% of the genome assembly. In total, the prediction of repetitive elements distributed 61.62 Mbp (18.18%) transposon DNA, 76.10 Mbp (22.46%) long terminal repeats (LTRs) and 5.19 Mbp (1.53%) unclassified and miniature inverted-repeat transposable elements (MITEs) (Fig. 4).

**Fig. 5:**
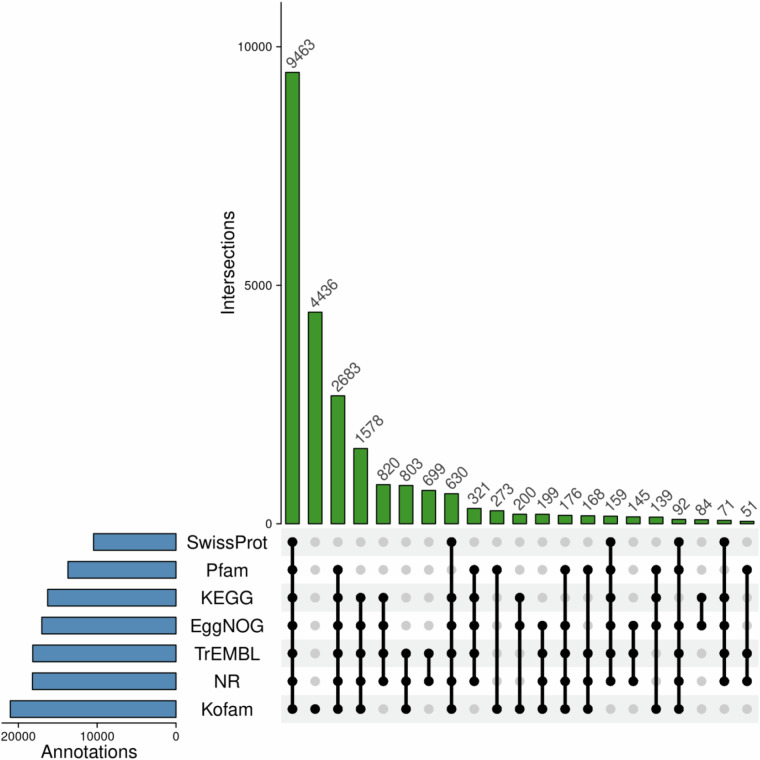
Upset plot comparing functional gene annotations across seven databases: KEGG, Swiss-Prot, EggNOG, NR, TrEMBL, Kofam, and Pfam.

The GC content and gene density were calculated in 500,000 nt windows for each chromosome using the GC.content function from the ape (v5.8) (https://github.com/emmanuelparadis/ape) package in the R environment. The highest average GC content was observed on chromosome 5 reaching 43.5%. The highest gene density (69) was observed on chromosome 8 between 26,500,000-27,000,000 bp (Fig. 5).

The cDNA long-read library was prepared for annotation of protein-coding genes. Briefly, total RNA was extracted from cryo-grinded thallus using RNeasy® Plant Mini Kit (Qiagen). The quantity of extracted RNA was measured with the Qubit^TM^ RNA HS Assay Kit. Next approximately 150 ng of total RNA was reversely transcribed to cDNA and amplified to prepare the cDNA library according to the protocol provided by ONT for cDNA PCR Sequencing Kit (SQK-PCS109). Adapter-ligated cDNA sequences were sequenced on the MinION flow cell (vR9.4.1, FC106D) with MinION Mk1C sequencing device.

The cDNA long reads were assembled *de novo* by RNA-Bloom (v2.0.1)^33^ to reconstruct 26,755 transcripts of *R. sorocarpa*. Next, the assembly transcriptome was implemented in the PASA pipeline (v2.5.3)^34^ to model complete and partial protein-coding genes. Simultaneously, *ab initio* predictions were carried out with the assistance of BRAKER (v2.0.4)^35^, GeneMark-ES/ET (v4.72)^36^ and AUGUSTUS (v3.3.3)^37^. Based on protein sequences of *Riccia fluitans* (PRJNA1158334), 30,615 potential gene annotations were predicted, respectively. Subsequently, a transcriptome-based set and two proteome-based predictions were integrated using EvidenceModeler (v2.1.0)^38^ with weight bias parameters and finally 27,626 protein-coding genes were obtained.

Next, unique transcripts were translated into protein sequences and functionally annotated against multiple protein databases using blastp (v2.12.0)^39^ and diamond (v2.0.15)^40^. The best matches of protein alignments were assigned to seven functional databases as follows: 10,456 had homologous with SwissProt^41^, 13,711 with protein families database (Pfam)^42^, 16,284 Kyoto Encyclopedia of Genes and Genomes (KEGG)^43^, 17,028 EggNOG-mapper^44^, 18,177 Translation of EMBL (TrEMB)^41^, 18,219 Non-Redundant Protein Sequence Database (http://www.ncbi.nlm.nih.gov/protein) and 21,030 Kofam (https://github.com/takaram/kofam_scan) (Fig. 5).

### Collinearity of Marchantiales

**Fig. 6:**
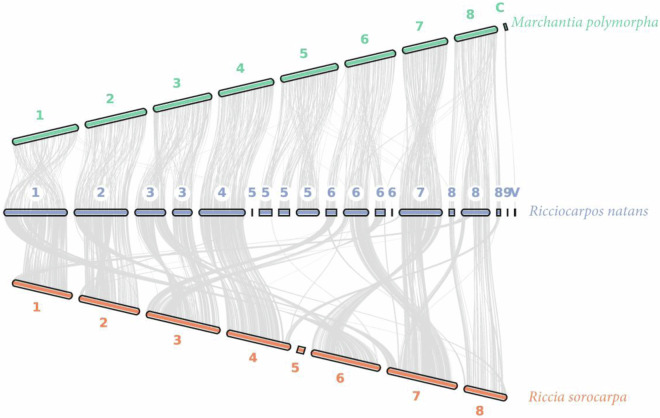
Synteny plot shows the differences and similarities between our assembly of the *Riccia sorocarpa* (orange) chromosomes and the assembly of *Ricciocarpos natans* (blue) and *Marchantia polymorpha* (green) by other authors. Grey line thickness reflects collinearity between species genomes.

Genome synteny between *R. sorocarpa*, *R. natans* and *M. polymorpha* were constructed by translating the longest isoforms of single-copy genes, utilizing GFF3 and BED format using JCVI software (v1.0.0)^45^. The collinearity between eight chromosomes of each Marchantiales revealed that chromosomes 1, 2 and 4 had highly conserved relationships between each species. Other chromosomes showed more complex and divergent synteny patterns. Interestingly, *R.sorocarpa* indicated a deep reduction of chromosome 5 (Fig. 6).

### Gene family evolution analysis

The trimmed alignment of single-copy proteins, along with the Newick format tree, was utilized to estimate the divergence times for the nodes in the phylogenetic tree using the *MegaX* program (v10.1.7)^46^ was used to estimate the divergence time for nodes in the phylogenetic tree. The species divergence time-points were calibrated by fossil data extracted from Bechteler *et al*.^47^, considering the divergence within *Syntrichia* genus [25 million years ago (mya)], *Sphagnum* genus (15 mya), between *S.ruralis* and *C.purpureus* (160 mya), *S.fallax* and *P. patens* (403 mya), *M. polymorpha* and *R. natans* (248 mya), and *M. polymorpha* and *P. patens* (455 mya). The estimated potential divergence time within *Marchantia* genus was established at 53.82 mya and between *R. natans* and *R. sorocarpa* at 174.8 mya. The dataset of 13 proteoms was evaluated by *Orthofinder* software (v2.5.4)^48^. The longest representants of each gene loci were extracted by AGAT software (v1.4.0)^49^. To reconstruct phylogenetic orthology inference of *R. sorocarpa* (27,626 proteins) and other fully annotated 12 Bryophytes species, the following translation of single-copy genes was used: 24,742 for *Anthoceros agrestis*^50^,30,425 for *Ceratodon purpureus*^51^, 23,428 for *Lunularia cruciate*^52^, 18,566 for *Marchantia palaceae*^53^, 17,944 for *Marchantia polymorpha* https://marchantia.info/, 32,926 for *Physcomitrium patens*^54^, 17,728 for *Ricciocarpos natans*^52^, 25,100 for *Sphagnum fallax*^55^, 25,227 for *Sphagnum magellanicum*^56^, 16,544 for *Syntrichia caninervis*^52^, 21,169 for *Syntrichia ruralis*^52^, and 27,467 for *Takakia lepidozioides*^57^.

**Fig. 7:**
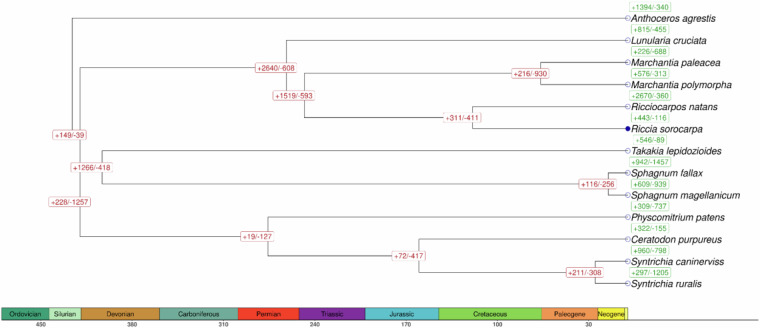
Divergence time tree of *R. sorocarpa* and 12 other species. The species divergence time was estimated using MegaX. Green and red numbers above each species’ name and divergence points reveal expansions (+) and contractions (−) of gene families.

Based on 18,261 single-copy orthogenes the gene family expansion and contraction analysis was done using the CAFE program (v5)^58^ (Fig. 7). Following the divergence of the *Riccia* genus from its common ancestor with *R. natans*, 960 gene families expanded while 798 gene families contracted. According to single-copy genes present in the majority of 13 species, the multi-sequence alignment (MSA) was performed. Next, the trimAl software (v1.4)^59^ cut the ambiguous positions within MSA and the phylogenetic tree was constructed using IQ-TREE 2 software (v2.2.0.3)^60^ (Fig. 7).

### DNA methylation calling

A previously generated BAM file was aligned using Dorado (v0.6.1) (https://github.com/nanoporetech/dorado) with our genome as a reference. It was then sorted and indexed employing Samtools (v1.12)^61^. The next step was to extract DNA methylation information leveraging modkit (v0.2.7.) (https://github.com/nanoporetech/modkit/releases). Methylations were qualified for further analysis when N_valid_cov > 20 and fraction_modified = 100. Next, methylations were divided into 5-methylcytosine (5mC) and 6-methyladenine (6 mA). A total of 404,506 methylated cytosines and 4,071 methylated adenines were identified (Fig. 4). Their percentage occurrence was calculated within a 500,000 window across the genome in the R environment. Terminal chromosome regions were treated as a single window when there were not enough nucleotides to fill the 500,000-base window. The highest percentage of methylated cytosines to cytosines within a window was observed on chr4 between 49000000-49131805 (1.09%), chr1 46500000-46876028 (0.99%), and chr5 4500000-5374985 (0.98%). The highest percentage of methylated adenines was observed on chr3 18500000-19000000 (0.021%), chr4 40500000-41000000 (0.02%), and chr3 49000000-49500000 (0.018%).

## Data Records

The Nanopore sequencing data used for genome assembly were deposited in the NCBI Sequence Read Archive database under BioProject PRJNA1130527^62^. The DNA long reads Nanopore sequencing data was deposited under SRR29669162 accession number. The Pore-C data was stored under accession numbers SRR29695115 and SRR29695116. The transcriptomic sequencing data were deposited in the SRA at NCBI accession SRR29695421^63^. The assembled genome and gene annotation can be found on NCBI assembly with the accession number JBJQOH000000000^64^.

## Technical Validation

The Q10 and Q15 of Nanopore sequencing data was greater than 96% and 68%, respectively. The Q10and Q15 of Pore-C sequencing data were greater than 95% and 52%, respectively. Genome completeness was confirmed by BUSCO (Benchmarking Universal Single-Copy Orthologs, v5.4.2)^65^ analysis, which identified 85.1% single-copy, 6.7% duplicated genes,1.6% fragmented and 6.6% missing in the eight longest scaffolds. To examine assembly integrity, the Nanopore trimmed sequences were checked using Minimap2 aligner (v2.17-r941)^66^ and Geneious Prime software (v2023.0.1, https://www.geneious.com). The 97.02% of chromosome regions had high completeness after contigs remapping procedure and described by median equal to 163. The Pore-C trimmed reads were also aligned with a high map rate of 84.56% to the manually curated *R. sorocarpa* genome. The evidence supports the reliability of the data, validating its use in subsequent analytical procedures.

## Usage Notes

The availability of this genome, the second near telomere-to-telomere assembly of a liverwort, provides a valuable resource for in-depth analysis of *Riccia sorocarpa*’s genetic diversity and will support further studies in adaptive evolution, phylogenomics, population genomics, and more. However, the low integrity of the raw DNA used for long-read sequencing has contributed to shorter ONT reads and reduced contig lengths. Future improvements to the genome assembly could be achieved with higher-quality DNA samples. The genome’s QV is reported to be above 35, which is considered moderate. Nevertheless, the application of the Pore-C method enables a satisfactory assembly of the genome. The process of organizing the genome at the chromosomal scale involved multiple iterations that integrated data from both long-read ONT sequencing and Pore-C. This comprehensive approach facilitated the accurate assembly of the *Riccia sorocarpa* genome.

## Data Availability

The guidelines for all bioinformatics tools employed in this study were adhered strictly. The software and code utilized are publicly available, with specific versions and parameters detailed in the Methods section. Our data compilation process did not involve any custom code.
